# Colonizing while migrating: how do individual enteric neural crest cells behave?

**DOI:** 10.1186/1741-7007-12-23

**Published:** 2014-03-26

**Authors:** Heather M Young, Annette J Bergner, Matthew J Simpson, Sonja J McKeown, Marlene M Hao, Colin R Anderson, Hideki Enomoto

**Affiliations:** 1 Department of Anatomy & Neuroscience, University of Melbourne, Melbourne 3010 VIC, Australia; 2 School of Mathematical Sciences, Queensland University of Technology, GPO Box 2434, Brisbane 4001 QLD, Australia; 3 RIKEN Center for Developmental Biology, Laboratory for Neuronal Differentiation and Regeneration, Kobe, Japan; 4 Division of Neural Differentiation and Regeneration, Department of Physiology and Cell Biology, Graduate School of Medicine, Kobe University, Kobe 650-0017, Japan

**Keywords:** Collective cell migration, Neural crest, Directional migration, Enteric nervous system

## Abstract

**Background:**

Directed cell migration is essential for normal development. In most of the migratory cell populations that have been analyzed in detail to date, all of the cells migrate as a collective from one location to another. However, there are also migratory cell populations that must populate the areas through which they migrate, and thus some cells get left behind while others advance. Very little is known about how individual cells behave to achieve concomitant directional migration and population of the migratory route. We examined the behavior of enteric neural crest-derived cells (ENCCs), which must both advance caudally to reach the anal end and populate each gut region.

**Results:**

The behavior of individual ENCCs was examined using live imaging and mice in which ENCCs express a photoconvertible protein. We show that individual ENCCs exhibit very variable directionalities and speed; as the migratory wavefront of ENCCs advances caudally, each gut region is populated primarily by some ENCCs migrating non-directionally. After populating each region, ENCCs remain migratory for at least 24 hours. Endothelin receptor type B (EDNRB) signaling is known to be essential for the normal advance of the ENCC population. We now show that perturbation of EDNRB principally affects individual ENCC speed rather than directionality. The trajectories of solitary ENCCs, which occur transiently at the wavefront, were consistent with an unbiased random walk and so cell-cell contact is essential for directional migration. ENCCs migrate in close association with neurites. We showed that although ENCCs often use neurites as substrates, ENCCs lead the way, neurites are not required for chain formation and neurite growth is more directional than the migration of ENCCs as a whole.

**Conclusions:**

Each gut region is initially populated by sub-populations of ENCCs migrating non-directionally, rather than stopping. This might provide a mechanism for ensuring a uniform density of ENCCs along the growing gut.

## Background

Neural crest cells are a transient migratory embryonic cell population. Previous imaging studies of cranial neural crest cells in *Xenopus* and chick embryos have revealed the organization of the cells as they migrate, the rules guiding their behavior and some of the molecular bases of the interactions [1,2]. In both species, the cells move as a collective from one location to another; in chick embryos, there is a “follow the leader” chain migration in which the spatial order of cells is retained [3-7]. In *Xenopus,* several concomitant behaviors have been identified including “run and tumble” in which phases of directional migration are interspersed with phases of small random movements, “mutual co-attraction” in which cells retain the same neighbors for long periods of time, and “contact inhibition of locomotion” in which cells polarize and disperse upon contact with each other [8-12].

A sub-population of vagal level (caudal hindbrain) neural crest cells migrates into and along the developing gut, and gives rise to most of the enteric nervous system [13-17]. The neural crest cells that colonize the gut migrate further than other embryonic cells because the gut is growing as the cells migrate [18,19]. The migration of ENCCs exhibits two important differences from neural crest cell populations that have been examined in detail previously. First, not only must some ENCCs migrate caudally to reach the distal regions of the gut, each gut region through which ENCCs migrate must be populated by ENCCs to ensure there is an even distribution of enteric neurons along the entire gut; this behavior has been termed “directional dispersion” [20]. In contrast, analyses of migrating cranial and trunk neural crest cells have been performed as the entire cell population migrates collectively from one location to another. Studies of ENCC migration to date have focused on the caudal advance of the ENCC wavefront [21-27]. ENCCs migrate in chains with high cell-cell contact [21,23,24,28], and so little is known about how individual ENCCs behave to ensure that all regions of the gut are also evenly populated with ENCCs. We had previously assumed that each gut region is colonized by sub-populations of ENCCs stopping as the wavefront of ENCCs moves caudally [23]. The migration of ENCCs is an excellent model to examine how individual cells behave in a population that both migrates directionally and populates regions along the migratory route.

A second important characteristic of the migration of ENCCs is that a subpopulation of ENCCs starts to differentiate into neurons that project neurites caudally along the same pathways that ENCCs are migrating [29,30]. The relationship between growing neurites and migrating ENCCs is still unclear, and it is unknown which leads the way at the migratory wavefront.

We used mice in which all ENCCs express the photoconvertible protein, KikGR [26] and so the behavior of individual ENCCs, their interactions with all other ENCCs, and their location with respect to the migratory wavefront, could be examined. We also examined the role of signaling via endothelin receptor type B (EDNRB) in migratory behavior. Detailed information about the migratory behavior of individual ENCCs at the cellular level is likely to provide clues as to the molecular mechanisms involved, and the data are also essential for the development of models of the colonization of the gut by ENCCs.

## Results

*Ednrb-hKikGR* mice, in which all ENCCs show KikGR fluorescence, were used [26]. KikGR is a photoconvertible protein that changes its emission from green to red following exposure to ultraviolet or violet light [4]. Individual or groups of KikGR cells in explants of embryonic gut were photoconverted from green to red, and then time-lapse imaging was performed in which three-dimensional image *z*-stacks were collected at two- to five-minute intervals on a confocal microscope. The majority of experiments were performed using explants of gut from E12.5 *Ednrb-hKikGR* mice because at this age, the ENCC migratory wavefront is in the mid-colon, and the colon is relatively straight [31]; as a result, the migratory wavefront of ENCCs could be imaged for over 24 hours. In contrast, at E11.5, the ENCC wavefront is difficult to define because of transmesenteric ENCCs, which migrate from the midgut across the mesentery into the proximal colon [26].

### Variability in speed and direction of migration of individual ENCCs at different distances from the migratory wavefront

**Figure 1:**
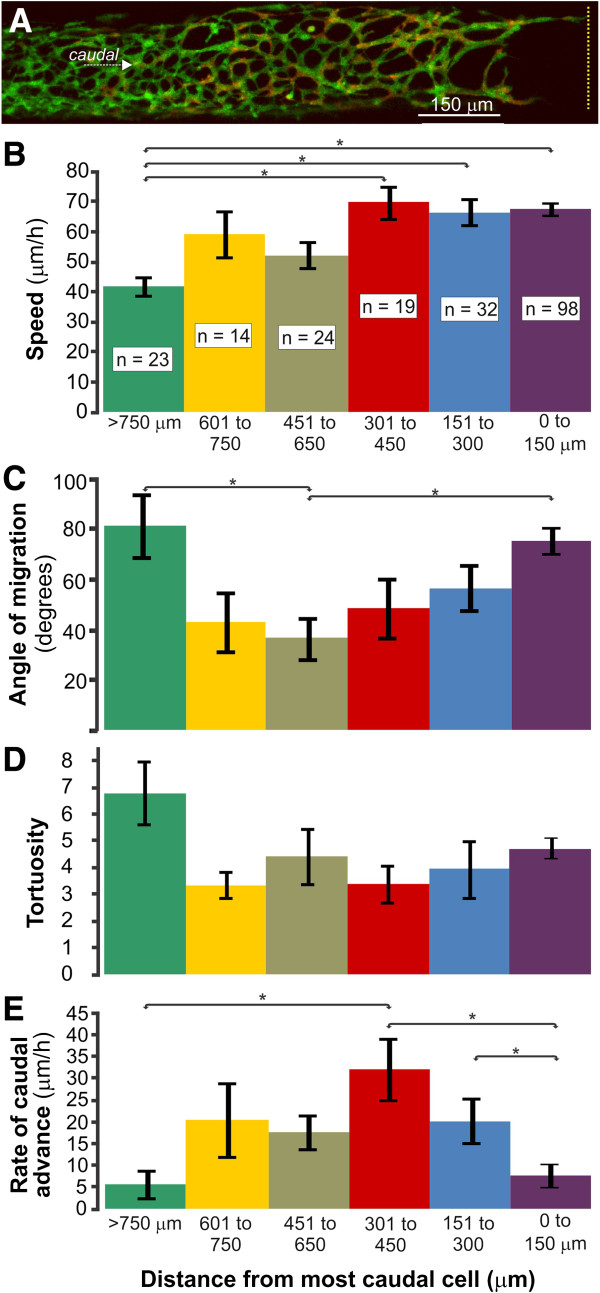
**Behavior of ENCCs at different locations. A**. An example of a preparation of E12.5 colon. The location of the most caudal cell is indicated with a yellow dotted line. **B**. The speed of ENCCs. ENCCs between 750 and 1,500 μm from the wavefront migrated significantly slower than those within 450 μm of the most caudal cell. **C**. The angle of migration between the beginning of end of imaging for each cell, relative to the long axis of the gut, which was defined as 0°. Only angles between 0 and 180° were used. **D**. Tortuosity; there were no significant differences between the tortuosity of ENCCs at different locations. **E**. Rate of caudal advance was defined as the longitudinal distance an ENCC advanced caudally during the imaging period regardless of the route (see Figure 2F). ENCC < 150 μm and > 750 μm from the most caudal cell showed the lowest rates of caudal advance. All data are shown as mean ± s.e.m. and were analyzed using ANOVAs followed by Tukey’s *post-*h*oc* tests. Asterisks indicate *P* < 0.05. ENCCs, enteric neural crest-derived cells.

ENCCs at defined locations with respect to the most caudal ENCC at the beginning of imaging were photoconverted; 210 photo-converted ENCCs within 1.2 mm of the most caudal ENCC in explants of E12.5 colon were tracked. Every cell that remained within the field of view for a minimum of two hours (mean imaging time per cell = 5.0 ± 0.02 hours (±s.e.m.); n = 50 explants) was included in this analysis, including cells with neuronal morphologies and solitary cells. To examine how ENCC behavior varies with location, speed (total distance traversed/time), overall direction of migration, tortuosity and rate of caudal advance of individual ENCCs were determined in 150 μm bins from the migratory wavefront for up to 1,200 μm rostrally (Figure 1).

**Figure 2:**
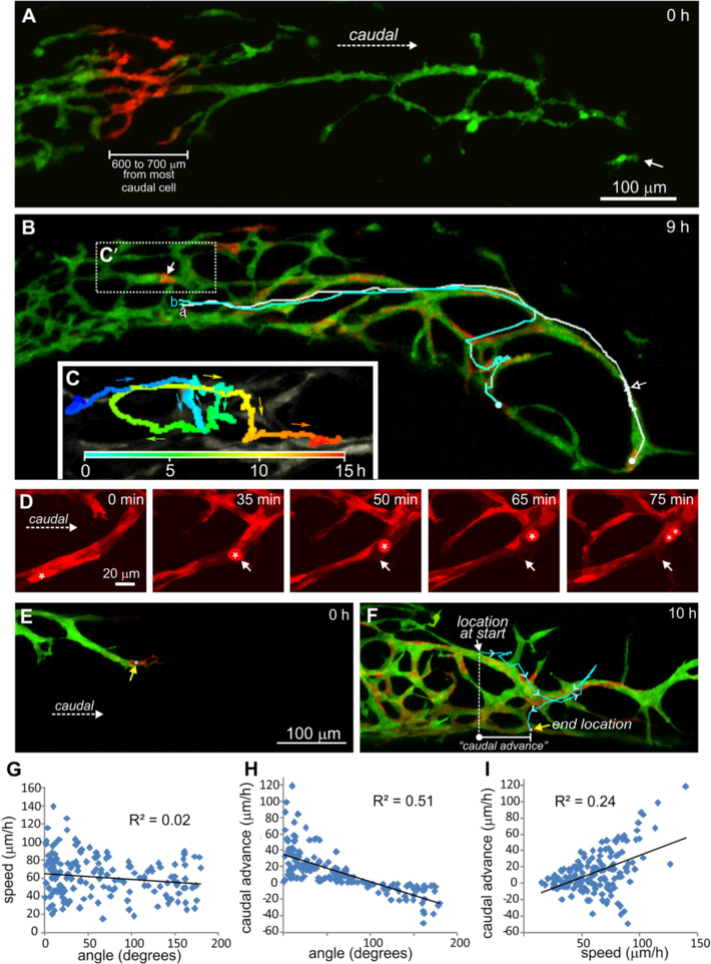
**Variability in caudal advance of individual ENCCs. A**. Cells that were 600 to 700 μm from the wavefront (white arrow) were photoconverted. **B**. Some photoconverted cells migrated caudally along existing strands with high speed and directionality (cells “a” and “b”, white and aqua tracks), and nine hours later, one of the cells (cell a) was very close to the wavefront; cell “a” migrated 980 μm in nine hours before going out of the field of view. Cell “a” (white track) also reversed direction and migrated rostrally for one hour, before migrating caudally again (white open arrow). **C**’. Another cell (arrow in **C**’) was imaged for 16 hours. **C**. This is an enlarged image of C’ with a color time track showing that it exhibited a complex, circular pathway; despite migrating at 70 μm/h, it advanced caudally only 140 μm after 16 hours. **D**. A red channel only showing a photoconverted cell (asterisk) that continues to advance caudally after it is first rounded up (arrow). **E**, **F**. Local leapfrogging at the migratory wavefront. **E**. The most caudal cell (yellow arrow) at the commencement of imaging was photoconverted. **F**. Ten hours later, the cell (yellow arrow) was > 200 μm behind the most caudal cell. Caudal advance was defined as the longitudinal caudal displacement of an ENCC during the imaging period regardless of its pathway; this cell advanced caudally only 70 μm in 10 h. The other red cells in this image are photoconverted cells that were out of the field of view (rostral) at the beginning. **G-I**. Correlations for the entire ENCC population shown in Figure 1 between speed and angle **(G)**, caudal advance and angle **(H)** and caudal advance and speed **(I)**. R^2^ is the correlation coefficient. A negative value for the rate of caudal advance means that the ENCC moved rostrally during the imaging period. ENCCs, enteric neural crest-derived cells.

The mean speed of ENCCs within 450 μm of the wavefront was significantly higher than those >750 μm from the wavefront (ANOVA followed by Tukey’s *post-hoc* test; Figure 1). However, the speeds of individual ENCCs were variable, and some ENCCs migrating within thick strands migrated at approximately 130 μm/h for over four hours (Figure 2A, B). “Migration” was defined as displacement of the center of the cell body a minimum of 20 μm every two hours. All photoconverted ENCCs analyzed migrated except one, which jiggled, but did not change location.

Overall migration direction was defined as the angle between the locations at the beginning and end of imaging relative to the long axis of the gut. Thus, a cell that was directly caudal to its starting position at the end of imaging had an overall migration direction of 0° regardless of its positions at intermediate times. For these analyses, we only used angles between 0° and 180° and did not distinguish between circumferential clockwise (90°) and counterclockwise (270°) migration around the gut. ENCCs that were <150 μm or >750 μm from the wavefront had higher overall migration angles than ENCCs 150 to 600 μm from the wavefront (ANOVA followed by Tukey’s *post-hoc* test; Figure 1C). Within thick strands oriented parallel to the long axis of the gut, most ENCCs migrated caudally (Additional file 1: Movie 1), and cells about to undergo cell division often continued to advance caudally even after rounding up for division (Figure 2D). However, ENCCs were also regularly observed that migrated in the opposite direction from most ENCCs in a strand, and a cell migrating in one direction could suddenly reverse direction (Figure 2B, cell a, 2F; Additional file 1: Movie 1). Furthermore, some cells followed very circuitous pathways (Figure 2C, cell c; Additional file 2: Movie 2). There was no correlation between speed and angle of migration (*R*^*2*^ = 0.02; linear regression for entire ENCC population; Figure 2G).

Tortuosity is a measure of the deviation from a straight line and is calculated by dividing the accumulated travel distance by the distance between the first and last points. There was no significant difference in the tortuosities of ENCCs at different locations (ANOVA; Figure 1D). Although tortuosity is a commonly measured parameter in migration studies, it does not take direction into consideration, so it is not very informative for understanding ENCC migration.

The rate of caudal advance was defined as the longitudinal caudal displacement/time of an ENCC, regardless of the route (Figure 2F). Individual ENCCs < 150 μm and > 750 μm from the most caudal cell had significantly lower rates of caudal advance than those 300 to 450 μm from the wavefront (ANOVA followed by Tukey’s *post-hoc* test; Figure 1E). Consequently, there is considerable local leapfrogging at the wavefront, and we did not observe any “pioneer” ENCCs that remained at the front of the ENCC population for more than two hours (Figure 2E, F).

### Gut regions behind the wavefront are primarily populated by ENCCs with low directionality

ENCCs with low rates of caudal advance will populate a region, rather than migrate into new regions. We examined whether the ENCCs that populate a region migrate at the same speed as other ENCCs but non-directionally, or whether they show similar directionality to other ENCCs but migrate slower. Linear regression analyses revealed that for caudal advance versus speed *R*^*2*^ = 0.24, while for caudal advance versus angle there was an inverse correlation with *R*^*2*^ = 0.51 (Figure 2H, I). Hence, low directionality appears to play a larger role than decreased speed in reducing caudal advance, and populating gut regions behind the wavefront.

**Figure 3:**
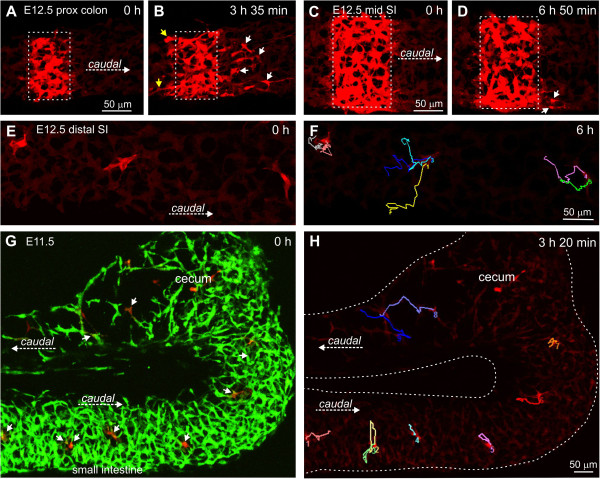
**Behavior of ENCCs rostral to the wavefront. A**, **B**. E12.5 proximal colon, approximately 1.1 mm behind the wavefront, red channel only. **B**. A total of 3.5 hours after photoconversion, many cells had migrated caudally (white arrows), and there was also some dispersal of cells rostrally for a short distance (yellow arrows). **C**, **D**. E12.5 mid-small intestine, red channel only. **D**. Although the cells were very dynamic, after seven hours, only a small number of cells (arrows) had migrated substantial distances longitudinally. **E**, **F**. E12.5 distal small intestine; three small groups of cells (seven cells in total) were photoconverted. **F**. Tracks of the seven cells showing that they migrated variable distances in variable directions. **G**, **H**. E11.5 gut. Individual cells (arrows) in a variety of locations were photoconverted **(G)**. H. Tracks of photoconverted cells overlaid on red channel only. Most cells in the pre-cecal gut were migratory, but often in a circumferential direction **(H)**. Pre-cecal ENCCs migrated slower (tracks are shorter) than post-cecal ENCCs. ENCCs, enteric neural crest-derived cells.

This conclusion was strengthened by the observation that ENCCs remain migratory in regions that have been long colonized by ENCCs. Cells in the E12.5 proximal colon, adjacent to the cecum (around 1 to 1.2 mm from the wavefront), commonly advanced caudally (Figure 3A, B). More orally, there was no mass caudally-directed movement of ENCCs within the mid and caudal small intestine of E12.5 mice (Figure 3C, D), which were initially colonized by ENCCs 24 to 36 hours previously. However, the ENCC network in the small intestine was very dynamic and most ENCCs migrated, but in variable directions (Figure 3E, F) and at variable speeds (mean ± s.e.m. = 25 ± 2 μm/h; n = 13). There was also extensive process extension and retraction. Migration of ENCCs in regions that had been colonized for up to 24 hours was also confirmed in the pre-cecal gut of E11.5 mice (Figure 3G, H).

### Solitary ENCCs follow an undirected random walk

**Figure 4:**
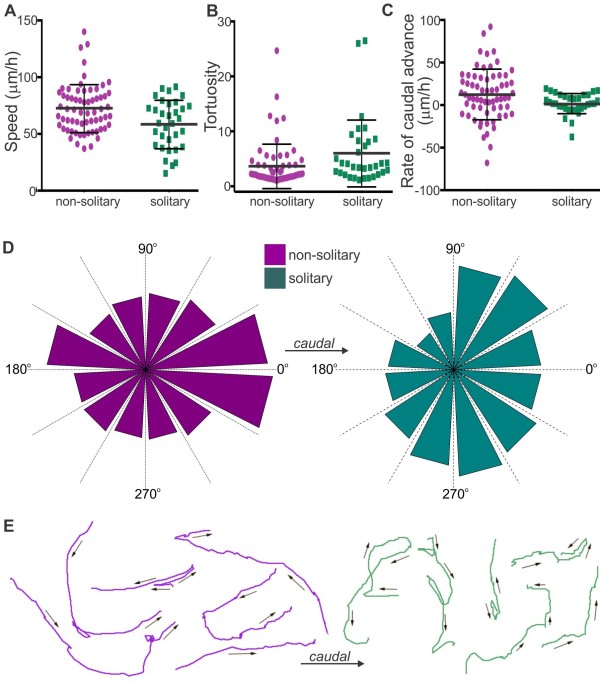
**Comparison of solitary and non-solitary cells within 150 μm of the wavefront.** Scatter plots for the speed **(A)**, tortuosity **(B)** and rate of caudal advance **(C)** of solitary (green) and non-solitary (magenta) ENCCs (mean ± 1 standard deviation). Compared to non-solitary cells, solitary cells migrated significantly slower (*P* = 0.003), exhibited higher tortuosity (*P* = 0.025) and had a lower rate of caudal advance (*P* = 0.049; n = 65 non-solitary ENCCs and 33 solitary ENCCs; unpaired *t* tests). **D**. Polar histograms showing the direction of migration measured at 10-minute intervals. The migration angles of solitary ENCCs were significantly different from those ENCCs in contact with other ENCCs (Watson’s *U*^2^ test for angles; n = 35 non-solitary and 22 solitary ENCCs, randomly chosen for analysis). **E**. Examples of tracks of non-solitary (magenta) and solitary (green) cells.

As described previously [22], transiently solitary ENCCs were commonly observed close to the migratory wavefront. Most solitary ENCCs were solitary for only 2 to 3 hours, but one ENCC was solitary for 14 hours. The behavior of ENCCs that were solitary for a minimum of two hours was examined to reveal the effect of cell-cell contact on migratory behavior. We first examined whether the trajectories of solitary ENCCs were mathematically consistent with a random walk, which is a pathway consisting of a succession of independent random steps [32]. A power-law was fitted to the time evolution of the square of the displacement for solitary ENCCs (n = 21, see Methods). Cells undergoing a random walk exhibit a linear mean square displacement in which the constant α = 1, whereas cells undergoing directed migration have a parabolic mean square displacement. For solitary ENCCs, *α* = 1.35 ± 0.58. As *α* is approximately 1, our data are consistent with the idea that solitary ENCC trajectories follow a random walk. In contrast, chains of ENCCs show an overall caudal advance. This was confirmed when the directions of migration (0° to 360° relative to the long axis of the gut) for solitary and non-solitary ENCCs, all within 150 μm of the most caudal cell, were determined at 10-minute intervals (note that the analyses in Figure 1B only determined the angle between the start and finish positions). The migration angles of solitary ENCCs were significantly different from those ENCCs in contact with other ENCCs (Watson’s *U*^2^ test for angles; [33]), with non-solitary ENCCs migrating caudally more frequently than solitary cells (Figure 4D, E). Moreover, although both solitary and non-solitary ENCCs exhibited variable behaviors, solitary ENCCs migrated significantly slower (*P* = 0.003, Figure 4A) and exhibited higher tortuosity (*P* = 0.025, Figure 4B; unpaired two-tailed *t* tests) than non-solitary ENCCs (all cells analyzed were < 150 μm from the most caudal cell). The difference in the rate of caudal advance only just reached statistical significance (12.4 μm/h for non-solitary ENCCs versus 1.7 μm/h for solitary ENCCs, *P* = 0.049, Figure 4C) due to large variability; in contrast to solitary cells, some non-solitary ENCCs migrated rostrally or caudally for considerable distances (Figure 4C). Of note, 38% of non-solitary ENCCs advanced caudally at > 20 μm/h whereas none of the solitary ENCCs (Figure 4C) advanced caudally at > 20 μm/h showing that a sub-population of non-solitary ENCCs is mainly responsible for migrating into new gut regions. In summary, although both solitary and non-solitary ENCCs close to the migratory wavefront exhibit very variable migratory behaviors, ENCCs in contact with other ENCCs migrate significantly faster and with a caudal bias, whereas the trajectories of solitary ENCCs are consistent with an undirected random walk.

### ENCCs do not retain their spatial order

**Figure 5:**
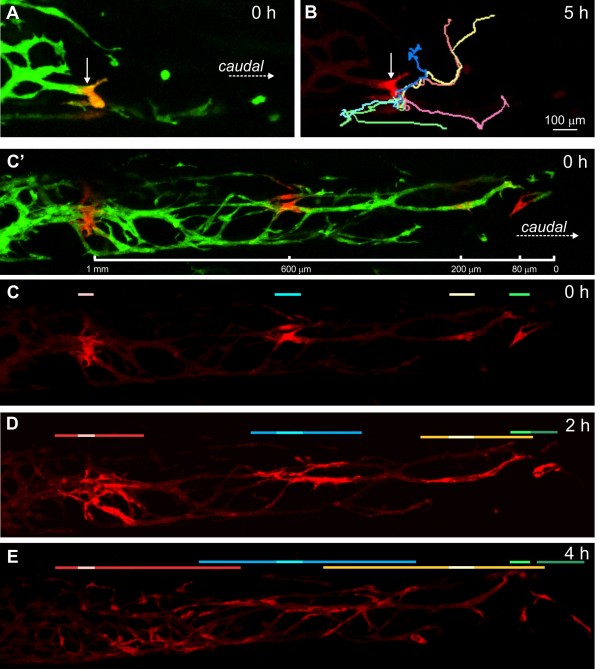
**Spatial order of cells within 1 mm of the migratory wavefront in the E12.5 colon. A**. The small group of cells (arrow) that were in contact with each other when photoconverted. **B**. Tracks of six of the cells in the subsequent five hours showing that they migrated in a variety of directions. **C-E**. A different E12.5 colon explant to show intermixing of ENCCs. **C**’. Red and green channels showing photoconversion of four groups of cells, which were 80, 200, 600 and 1,000 μm from the most caudal cell at the commencement of imaging. **C**. Red channel only showing the extents of initial locations of each group, which are marked by the pale green, yellow, blue and pink bars. **D**, **E** The spatial extents of cells two hours **(D)** and four hours **(E)** later are shown by the darker green, yellow, blue and red bars. The darker lines indicate the furthest extent of dispersal of cells from each group, not the number of cells; more cells dispersed caudally than rostrally from each group of photoconverted cells. ENCCs, enteric neural crest-derived cells.

Cranial neural crest cells in chick and zebrafish maintain the same neighbors for extensive periods of time and so the spatial order of cells is largely retained [4,10,34]. We examined the spatial order of ENCCs within 1 mm of the most caudal cell in the E12.5 colon. When small groups of ENCCs (two to eight cells) were photoconverted, they migrated in variable directions and it was very rare for any of the cells to have retained contact with their original neighbors after two hours (Figure 5A, B). There was considerable mixing of cells in different locations (Figure 5C-E; Additional file 3: Movie 3). Thus, although there is no large scale longitudinal mixing of ENCCs in the small intestine (see Figure 3C, D), within 1 mm of the wavefront, the spatial order of cells is not retained.

### Interactions between ENCCs are mainly adhesive

Detailed observations of the interactions between ENCCs are described in Additional file 4: Figures S1 and S2. In brief, ENCCs at the fronts of chains extended filopodia and lamellipodia in a variety of directions into unoccupied regions (Additional file 5: Movie 4) and ENCCs within chains extended processes within and outside of chains (Additional file 4: Figure S1). There was no significant difference between the protrusive activities of ENCCs that were 200 to 400 μm (where new ENCC network is still forming) and 500 to 800 μm (where the network is largely established) from the wavefront (Additional file 4: Figure S1). The predominant type of interaction between chains of ENCCs was adhesive interactions (Additional file 4: Figure S2; Additional file 5: Movie 4).

### ENCCs migrate in close association with neurites but neurites show higher directionality

#### ***Neurite behavior***

A sub-population of ENCCs starts to express pan-neuronal markers soon after they enter the gut [29]. Neuron-like cells can occur within 100 μm of the wavefront, and possess a single, persistent caudally-directed neurite [30]. In agreement with our earlier study [26], neurons migrated slower than non-neuronal ENCCs (neurons: 43 ± 4 μm/h, n = 18; non-neuronal cells: 64 ± 3 μm/h, n = 76; unpaired two-tailed *t* test; *P* <0.05; neurons and non-neuronal ENCCs included in this analysis were 200 to 800 μm from the wavefront. The cells are also included in the data shown in Figure 1).

**Figure 6:**
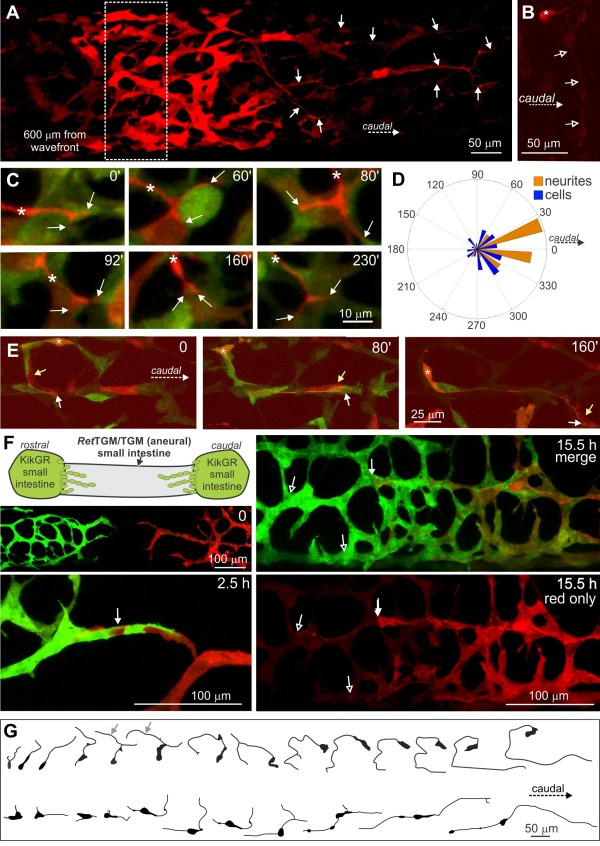
**Behavior of neurites. A**. A total of 5.5 hours following photoconversion of the entire diameter of the E12.5 colon (hashed box), 600 μm from the wavefront. Numerous neurites (white arrows) were present on the caudal side. More cells migrated caudally than rostrally. **B**. A neuron with a circumferentially projecting neurite in the E12.5 proximal colon. **C**. Neurites do not normally explore outside of the ENCC network. Growth cone of a neurite in the E12.5 colon, which was often bifurcated (arrows), and only explored within the ENCC network. The main shaft of the neurite is marked by an asterisk. **D**. Polar frequency histogram showing the direction of neurite advance (n = 9) and migrating ENCCs (n = 6) measured at 10-minute intervals. The directions of neurite advance were significantly different from ENCC migration direction (Watson’s *U*^2^ test for angles). **E.** Close association between a growing neurite and an individual ENCC. The growing tip (white arrow) of the neurite (orange) initially extends circumferentially and then the neurite extends caudally. It grew in close association with a photoconverted (red) ENCC (yellow arrow) for over 2.5 hours. The neuron cell body (asterisk) also migrates. **F**. Segments of *Ednrb-hKikGR* small intestine were placed at both ends of an explant of *Ret*^TGM/TGM^ small intestine, which lacks ENCCs. When ENCCs from the two populations were approximately 100 μm apart, the rostrally migrating population was photoconverted (time = 0). When the two populations collided, chains of intermingled cells formed (arrow, 2.5 h). Both populations then ceased to advance, although red neurites (open arrows) continue to grow rostrally for over 15 hours, well beyond the most rostral ENCC (arrow). **G**. Tracings of two ENCCs in the initial stages of neurite projection that were imaged for 12 hours (top cell) and 7 hours (bottom). ENCCs, enteric neural crest-derived cells.

When groups of ENCCs spanning the entire width of the caudal small intestine or colon were photoconverted, labeled neurites were only observed on the caudal side (Figure 6A; Additional file 6: Movie 5). However, in the longer established ENCC network of the E12.5 small intestine and proximal colon, neurites that projected circumferentially were also commonly observed following photoconversion of small groups of ENCCs (Figure 6B).

The growth cones at the tips of growing neurites only explored within the ENCC network (Figure 6C). This was in contrast to the lamellipodia and filopodia of ENCCs, which commonly extended outside of the ENCC networks (see Additional file 4: Figure S1). We observed several examples where a growth cone advanced caudally in concert with a particular ENCC for over two hours (Figure 6E). The rate of caudal advance of neurites (measured as the longitudinal distance advanced caudally regardless of the pathway) was 39 ± 5 μm/h (n = 8), which is similar to the rate at which the ENCC population advances caudally (35 to 40 μm/h) [21,23,35]. We compared the directionality of neurite extension with that of migrating ENCCs; the neurites were 200 to 800 μm from the wavefront, and the six ENCCs analyzed were all from one preparation and were in a group that was 500 μm from the wavefront. Neurites showed more directional (longitudinal) advance than the ENCCs (Figure 6D; Watson’s *U*^2^ test for angles; [33]).

When ENCCs are introduced into explants of small intestines lacking ENCCs (from *Ret*^TGM/TGM^ mice), they migrate equally well rostrally as they do caudally [36]. In the current study we used a similar co-culture system to examine the behavior of neurites in a rostrally-migrating population of ENCCs. Segments of small intestine from *Ednrb-hKikGR* mice were placed at the rostral and caudal ends of explants of aneural small intestines from *Ret*^TGM/TGM^ mice (Figure 6F). The ENCC population migrating rostrally was photoconverted from green to red just prior to encountering the caudally-migrating population (Figure 6F). The caudally-migrating (green) and rostrally-migrating (red) ENCCs adhered to each other to form chains of intermingled cells. It was not possible to analyze the effects of collisions between ENCCs migrating in opposite directions on individual cell behavior because high levels of cell-cell contact prevented us from obtaining pre-collision data for individual ENCCs. After forming a network, the red and green ENCCs continued to migrate for at least 22 hours, but non-directionally (Figure 6F). In contrast, red neurites continued to grow rostrally (Figure 6F). We were unable to visualize whether any of the red ENCCs also projected neurites caudally.

#### ***Initial extension of neurites***

Most neuron-like ENCCs in the E11.5 and E12.5 gut project caudally [37,38]. Four of the movies of the E12.5 colon contained photoconverted ENCCs undergoing neuritogenesis. In all cases, the neurite formed from a thin process that continued to elongate. In two out of four cells, the neurites did not initially emerge from the caudal side of the cell bodies, and in three out of four cells, the neurites did not initially project caudally, but first projected rostrally or circumferentially prior to projecting caudally (Figure 6G). These data suggest that most individual neuron precursors are not polarized prior to extending a neurite.

#### ***Neurite-ENCC interactions at the migratory wavefront***

**Figure 7:**
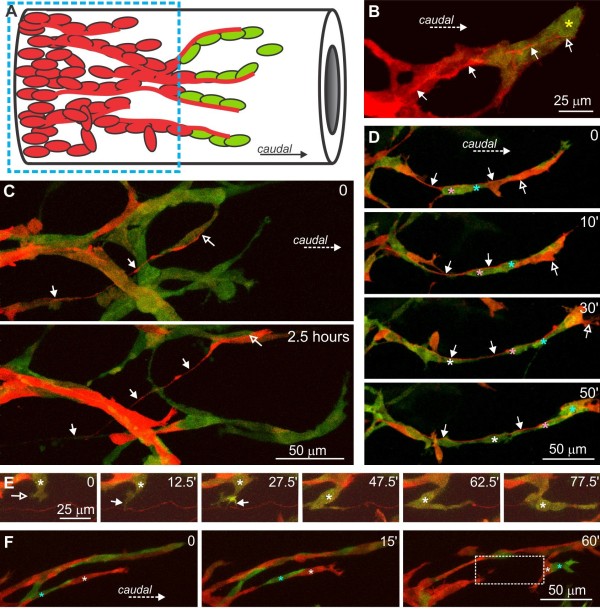
**Interactions between neurites and migrating ENCCs at the migratory wavefront. A**. A diagram showing photoconversion of all ENCCs, except the most caudal ENCCs, which results in photoconversion of the caudally-projecting neurites. **B**. The tip (open arrow) of a red, caudally-projecting neurite (arrows) is located at the back of the most caudal cell (yellow asterisk). **C**. The tip (open arrow) of a red neurite (arrows) advances slightly behind a migrating ENCC. Even though the neurite advanced in association with migrating ENCCs, 2.5 hours later, there were few ENCCs associated with some sections of the neurite (arrows). **D**. The tip (open arrow) of a caudally projecting neurite (arrows) is just rostral to the most caudal group of ENCCs, but it acts as a substrate for ENCCs (blue, pink and white asterisks) just behind the most caudal ENCCs. **E**. Adhesive interactions between a neurite (red) and a migrating ENCC. **F**. Not all chains of ENCCs have neurites associated with them. Two ENCCs (white and blue asterisks) extend caudally as a chain. After they lose contact with the ENCCs behind and join another chain, no neurites are visible (boxed area). ENCCs, enteric neural crest-derived cells.

Neurites are closely associated with ENCCs, but it is currently unclear whether neurites or ENCCs are the lead elements in the caudal migration. We photoconverted every ENCC in the E12.5 mid-colon, except for those within approximately 100 μm of the most caudal cell; photoconverted (red) KikGR protein from nerve cell bodies > 100 μm from the wavefront rapidly spread into the caudally directed neurites among green ENCCs (Figure 7A). Neurites were commonly observed in association with ENCCs at the migratory wavefront, but they were usually located just behind, or a few cells behind, the most caudal ENCC in a chain (Figure 7B,C). If a neurite were present close to the wavefront, migrating ENCCs usually used the neurite as a substrate to advance caudally (Figure 7D, E; Additional file 7: Movie 6). However, some ENCCs extended filopodia and lamellipodia away from neurites, and ENCCs were observed to detach from neurites. We also observed chains of migrating ENCCs that broke to reveal that there was no accompanying neurite (Figure 7F). In addition, we observed neurites extend in association with ENCCs, but the ENCCs then migrated away, or migrated rostrally back along the neurite, leaving a neurite without accompanying ENCCs (Figure 7C). In summary, ENCCs lead the way with neurites often located just rostral to the leading ENCCs; these neurites generally act as substrates for the following ENCCs. However, chains of ENCCs can extend without an accompanying neurite. We did not observe any neurites extending caudally in advance of migrating ENCCs.

#### ***Relationship between ENCC migration direction and neurite orientation well behind the wavefront***

**Figure 8:**
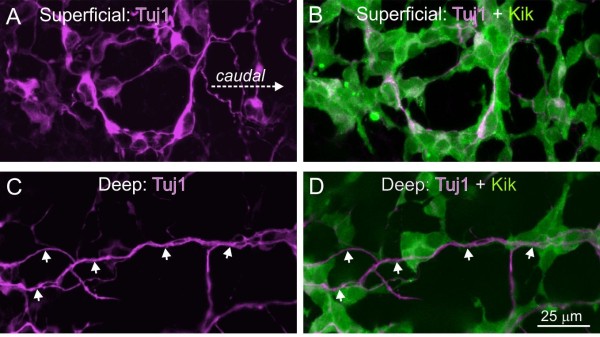
**Thick bundles of longitudinally-oriented neurites are deeper (closer to the lumen) than the majority of ENCCs.** Single optical sections of whole-mount preparations of E12.5 small intestine of an *Ednrb-hKikGR* mouse that was also immunostained using an antibody to Tuj1 (magenta). **A**, **B**. Single optical section at a focal plane in which there is the highest density of ENCCs. There is a plexus of Tuj1+ cell bodies and neurites with no obvious orientation. **C**, **D**. At the focal plane of the longitudinally-oriented neurite bundles, the density of ENCCs is lower. ENCCs, enteric neural crest-derived cells.

There are prominent, longitudinally-oriented bundles of neurites in regions long colonized by ENCCs [38,39]. Here, we reported the paradoxical observations that migrating ENCCs often use neurites as substrates, and that ENCCs well behind the wavefront migrate, but mostly circumferentially (see above). Examination of fixed preparations of small intestinea from E12.5 *Ednrb-hKikGR* mice that had been immunostained using the neurite marker, Tuj1, revealed a fine neurite network without any detectable orientation at the same focal plane as most of the ENCCs (Figure 8A, B). Thick bundles of longitudinally-oriented neurites were present deeper (closer to the circular muscle layer) than the majority of ENCCs (Figure 8C, D).

### Inhibition of EDNRB signaling primarily affects individual ENCC speed

**Figure 9:**
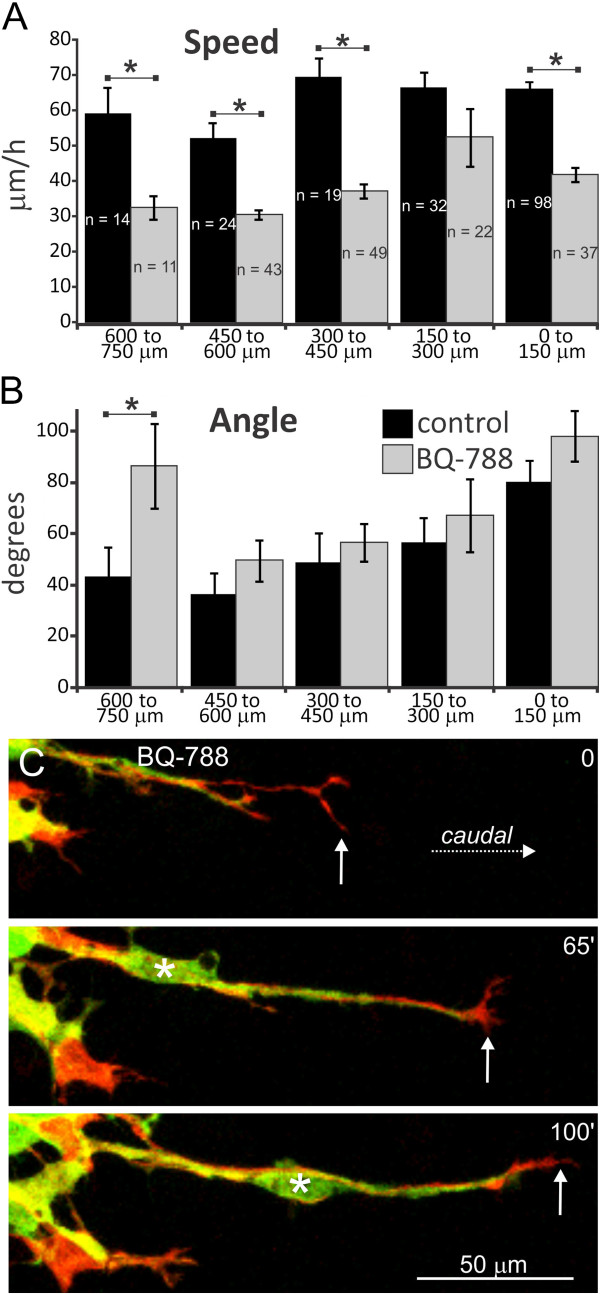
**Inhibition of EDNRB signaling primarily affects the speed of ENCC migration. A**, **B**. The speed and angle of migration (mean ± s.e.m.) of tracked control ENCCs (these are the same data as shown in Figure 1) and ENCCs in explants exposed to the EDNRB antagonist, BQ-788 (20 μM). **A**. ENCC speed in explants exposed to BQ-788 is significantly lower than in control explants, except for ENCCs that were 150 to 300 μm from the migratory wavefront (*t* tests). **B**. The angle of migration between the locations at the beginning and end of imaging for each ENCC, relative to the long axis of the gut. Only angles between 0° and 180° were used. The angles of ENCCs exposed to BQ-788 were not significantly different from controls except for ENCCs that were 600 to 750 μm from the wavefront, where BQ-788-treated ENCCs migrated at a higher angle than control ENCCs. **C**. E12.5 colon explant exposed to BQ-788. The growth cone (arrows) of a neurite is in advance of the most caudal ENCC cell body. An ENCC cell body (asterisk) uses the neurite as a substrate. EDNRB, endothelin receptor type B; ENCCs, enteric neural crest-derived cells.

The caudal advance of ENCCs along colonic explants is retarded by the EDNRB inhibitor, BQ-788 [40-42]. We examined whether the delay is due to individual ENCCs migrating slower and/or less directionally. ENCCs in colonic explants from E12.5 *Ednrb-hKikGR* mice exposed to BQ-788 (20 μM) migrated significantly slower than control ENCCs, except those that were 150 to 300 μm behind the wavefront (Figure 9A; two-tailed *t* tests, *P* < 0.05, except for 150 to 300 μm ENCCs where *P* = 0.13; 162 cells were tracked in 12 colon explants exposed to BQ-788; the control data are the same as those shown in Figure 1B, C). Four ENCCs were stationary in the presence of BQ-788. Although EDNRB signaling is known to influence the rate of neuronal differentiation of ENCCs [43,44], the decrease in migration speed of individual ENCCs exposed to BQ-788 for 10 hours was not associated with any detectable change in the proportion of Hu + neurons (Additional file 4: Figure S3). There was no significant difference in the direction of migration between control and BQ-788-exposed ENCCs except for ENCCs that were 600 to 750 μm from the wavefront (two-tailed *t* tests; Figure 9B). As in control mice, BQ-788-treated ENCCs that were 0 to 150 μm from the most caudal cell showed lower directionality than ENCCs further rostral, and there was no correlation between angle and speed (*R*^*2*^ = 0.01; linear regression for entire cell population). These data show that inhibition of EDNRB signaling primarily affects the speed at which individual ENCCs migrate. Unlike control explants, in some BQ-788-treated explants, we observed neurites transiently in advance of ENCCs (Figure 9C).

## Discussion

Most migratory cell populations that have been studied in detail to date migrate as a collective from one location to another. In contrast, the formation of an enteric nervous system along the entire gut requires that some ENCCs advance caudally while others populate each region, which has been termed “directional dispersion” [20]. In this study we showed that a characteristic of ENCCs is their variability in migratory behavior, particularly directionality. Most ENCCs appeared to migrate according to their own individual program while maintaining contact with other ENCCs - for example, ENCCs did not retain their neighbors and cells migrating in opposite directions within the same chain were commonly observed. We suggest that variability in ENCC behavior, but with an overall caudal bias, results in the colonization of each gut region as well as the caudal advance of some ENCCs. Despite the variability, our study shows that ENCCs behave differently when solitary compared to groups, and therefore meet the definition of collective migration [45]. For the majority of time, the behavior of ENCCs did not in general conform to the types of migratory behaviors described previously for neural crest populations that migrate as a collective from one location to another [4,8-10]. However, some behaviors described for other neural crest populations, including contact inhibition of locomotion, were exhibited occasionally, and we cannot rule out the possibility that non-predominant behaviors play an important role in ENCC migration.

### Colonization of all regions of the gut with ENCCs

The ENCCs that populate a gut region are those that show the lowest rate of caudal advance. Our data show low ENCC caudal advance is more highly correlated with low directionality than with low speed. Thus, the gut is colonized primarily by ENCCs migrating non-directionally, although ENCCs migrating slower than the rest of the population also contribute to gut colonization. Furthermore, it was not possible to separate the ENCC population in the E12.5 colon into two distinct groups - those that advanced caudally and those that did not.

A recent imaging study using biosensors revealed that ENCC migration speed was positively correlated with Rac1 and Cdc42 activities and inversely correlated with protein kinase A (PKA) activity [46]. Hence the activities of Rac1, Cdc42 and PKA are likely to play a role in determining the rate at which an ENCC advances caudally by regulating ENCC speed. We showed that directionality is also an important determinant in whether an ENCC will advance caudally, and that there was no correlation between direction and speed. Future live imaging of signaling molecule activities in ENCCs are required to reveal whether migration direction is correlated with the activity of signaling molecules at particular subcellular locations within an ENCC, as has been performed in living *Xenopus* cranial neural crest cells [9] and fixed ENCCs [47].

Unexpectedly, most ENCCs were still migratory in regions of the gut that had been colonized for over 24 hours, although they migrated slower than ENCCs closer to the wavefront. Many ENCCs in long-colonized regions migrated circumferentially. This was also surprising as there are prominent longitudinally-oriented neurite bundles in colonized regions of the gut [38,48], and we showed that ENCCs advancing caudally often use neurites as a migratory substrate. However, we also demonstrated that the thick longitudinally-oriented neurites are located deeper than the un-oriented neurite network associated with most ENCCs. In *Ednrb* mutant mice where ENCC migration is delayed, the E14.5 mid-colon is colonized by ENCCs migrating circumferentially from a dorsal mesenteric strand [40]. Combined the data suggest that as the gut matures, it favors the circumferential migration of ENCCs. The mechanisms promoting circumferential migration remain to be identified, but are unlikely to be related to the vasculature as the blood vessel network in the mouse intestine is mostly associated with the mucosal crypts and the submucosa [23,49], whereas the circumferential migration reported in the current study is mainly in the outer mesenchyme where the myenteric plexus will later form. Furthermore, circumferentially migrating ENCCs migrated within the existing ENCC network.

Consistent with our earlier study [23], we found that the shapes of the ENCC networks first laid down are retained over time. As we now show that most ENCCs continue to migrate after they colonize each gut region, we propose that the ENCC network is maintained by a scaffold of un-oriented neurites that is present at the same level as the majority of ENCCs. In the mature gut, myenteric ganglia are long structures that are oriented parallel to the circular muscle layer. In contrast, the clusters of ENCC present early in ENS development are not oriented in any particular direction and are dynamic in their composition. Hence, major reorganization of the ENCCs occurs prior to mature ganglion formation.

During development of the lateral line in zebrafish, cells migrate collectively along the body of the embryo and deposit cell clusters called rosettes at discrete locations; these are the progenitors of mechanosensory organs [50]. A recent study showed that the deposition of a sub-population of cells to form rosettes during the migration of the collective involves the formation of microtubule-dependent adherens junctions [51]. The formation of mechanosensory organs at distinct intervals in the zebrafish lateral line is, however, different from the directional dispersion of ENCCs, as ENCCs are dispersed evenly along the entire length of the gut. Like ENCCs, other neural crest-derived cell populations, such as Schwann cells and melanocytes, evenly populate structures as they migrate distally. The migratory behavior of these cell types has not yet been examined in detail, but a study of intact skin explants from E14.5 mice showed that melanoblasts remained migratory in regions already colonized [52]. Unlike ENCCs, however, melanocytes are found as solitary cells that exhibit repulsive interactions upon contact with other melanocytes and consequently maintain individual territories [52].

### Frontal advance

Earlier studies have shown that ENCCs at the migratory wave front are mainly responsible for populating uncolonized regions of the gut; this process has been termed “frontal advance” [22,26,27]. Our data are in agreement with this model but provide some refinements. First, we show that there is considerable local leapfrogging of ENCCs at the wavefront because the most caudal cells on average show low directionality and hence low caudal advance. Thus, within the frontal advance zone, ENCCs do not retain their spatial order. Previous studies in which small regions of mesenchyme in explants of chick gut were replaced with quail gut mesenchyme suggested that the ENCC frontal advance zone is only around 200 to 300 μm long [27]. Our data show that ENCCs advancing caudally are not found in a distinct zone with definable boundaries - ENCCs located 300 to 450 μm from the most caudal cell showed the highest average rate of caudal advance, and the probability that an ENCC would advance caudally decreased caudo-rostrally thereafter so that in the small intestine (> 1.2 mm from the wavefront), there was no significant caudal advance even though most of the cells were still migratory. It is likely that the length of the ENCC frontal advance zone varies with age and species.

### ENCCs at the migratory wavefront show low directionality

Cranial and trunk neural crest cells in the chick migrate in chains with clear leading cells [3,53,54]. Even excluding solitary cells, our data show that the extreme wavefront ENCCs migrate with lower directionality than ENCCs that are 150 to 600 μm from the wavefront. Druckenbrod and Epstein (2007) [22] tracked individual ENCCs in the chick gut that had been labeled with GFP by electroporation and, consistent with the current study, ENCC behavior was shown to be variable, and to vary at different distances from the wavefront. However, ENCCs within 200 μm of the wavefront were observed to show the most directional migration [22]. Hence, there may be some species differences in the behaviors of ENCCs.

We were unable to shed light on the unresolved question of why chains of ENCCs advance into uncolonized regions. Unlike follow-the-leader chain migration of neural crest cells in chicks [54], our study showed that the protrusive activity of ENCCs does not vary with location. Combined, the data are consistent with the idea that ENCCs cannot detect the polarity of the gut, but preferentially migrate into areas of low ENCC density while retaining contact with other ENCCs.

### Role of ENCC-ENCC contact

Our study shows that although ENCCs generally do not maintain the same neighbors for long, there are strong transient adhesive interactions between neighboring ENCCs. The molecular bases of the adhesive interactions between ENCCs are not completely understood but L1CAM and β1 integrins play roles [55-59]. Despite the strong adhesive interactions, ENCCs that are transiently solitary are frequently observed at the migratory wavefront [22]. For follow-the-leader chain migration of cranial and trunk neural crest cells, it has been suggested that cells in a chain follow a path of lesser resistance through the ECM and, therefore, migrate faster than solitary cells [7]. Consistent with this idea, the speed of solitary ENCCs was significantly slower than ENCCs in chains. Furthermore, ENCCs in chains migrated more directionally than solitary ENCCs suggesting that cell-cell contact is essential for ENCCs to respond to environmental cues as has been shown for cranial neural crest cells in *Xenopus*[9] (see below).

### Role of EDNRB signaling

Endothelin-3 (EDN3) acting at EDNRB receptors expressed on ENCCs is essential for the normal colonization of the gut by ENCCs [60-63]. Interpretation of the migratory behavior of ENCCs in *Ednrb* null mutant mice is difficult because the entry of neural crest-derived cells into the gut is delayed and thus the gut environment through which ENCCs migrate is older than in wildtype mice [40]. Our data, using a pharmacological inhibitor of EDNRB, reveal that EDNRB signaling primarily promotes migration speed, rather than directionality. EDNRB signaling is likely to affect ENCC migration via PKA activity [46,64]. Although it is well established that EDNRB signaling inhibits ENCC differentiation [65], we showed that exposure of explants to the EDNRB inhibitor in the timespan of our experiments was not sufficient to result in any detectable changes in neuronal differentiation. Thus the effect of inhibition of EDNRB signaling on ENCC migration cannot be entirely due to an enhancement of neuronal differentiation and our data therefore support the conclusions of a previous study [40] showing that EDNRB signaling has a direct effect on ENCC migration, as well as an indirect effect via its influence on differentiation. It will be interesting to determine whether the delay in the caudal advance of the ENCC population in other models of Hirschsprung disease is due to perturbed speed or directionality of individual ENCCs.

### Longitudinal growth of axons

We showed that neurites within the E12.5 colon advance caudally with overall high directionality, implying that neurites can detect the longitudinal axis and the polarity of the gut. However, when ENCCs were introduced into the caudal end of explants of aneural small intestine, some neurites grew orally. Our data are consistent with the idea that neurites use a property of the gut tube to detect the longitudinal axis of the gut, but the mechanism regulating the direction of neurite growth remains to be determined. We have previously suggested that the direction of neurite growth might be determined by the direction of ENCC migration [30]. However, in the current study, when the orally projecting neurites in the co-cultures encountered a population of ENCCs migrating anally, they continued to grow orally. Furthermore, following pharmacological inhibition of EDNRB signaling, neurites were observed in advance of ENCCs, showing that ENCCs are not required for longitudinal neurite extension. In control explants, the growth cones of the growing neurites did not explore outside of the ENCC network, and thus it appears highly unlikely that neurites normally navigate along longitudinally-oriented structural components of the gut wall, outside of the ENCC network. Mature enteric neurons also include subtypes that project circumferentially or orally [66]. Some circumferentially projecting neurons were observed at E12.5, but only in the small intestine and proximal colon.

### Role of neurites in ENCC migration

Sacral neural crest cells migrate into the hindgut along the axons of extrinsic neurons [15]. Neurites (from intrinsic neurons) are present in close apposition to chains of vagal ENCC, but it was previously unclear whether neurites or ENCCs lead the way; in fixed tissue the most caudal structure is most commonly an ENCC, but it is also not unusual to observe neurites in advance of ENCCs [67]. Our data show that ENCCs lead the way down the gut, with neurites following a short distance behind. Furthermore, the growth cones of neurites only explored within the ENCC network, and as a result, neurites always advanced in concert with chains of ENCCs. Sometimes ENCCs were observed to reverse direction and migrate back along a neurite or to migrate away from a neurite, which explains why neurites are sometimes observed caudal to the most caudal ENCC in fixed tissue. Our data also show that neurites act as substrates for migrating ENCCs. Although neurites are not required for the extension of chains of ENCCs into unpopulated regions, it is possible that longitudinally oriented neurites facilitate the directional migration of ENCCs towards the wavefront. Previous studies have provided evidence both for and against an essential role for neurites in ENCC migration. Humans with mutations in the gene encoding kinesin binding protein (KBP) have Hirschsprung disease [68], and studies in neuronal cell lines, primary cortical neurons and zebrafish have shown that the main role of KBP appears to be in neurite formation [69-71]. On the other hand, mutant mice with defects in the longitudinal projections of neurites do not exhibit any delay in ENCC migration [38] showing that longitudinal neurite projections are not essential, or that only a small number of longitudinally oriented neurites is necessary, for normal ENCC migration. To reveal the role of neurites in ENCC migration conclusively, future studies are required in which neurite formation is blocked in differentiating neurons.

### Individual ENCC are unable to detect gradients of morphogens along the gut

A number of molecules that influence the migration of ENCC *in vitro* are found in gradients along the gut [72-74]. For example, GDNF is chemoattractive to ENCCs in cultured explants, and *Gdnf* is expressed in a spatial and temporal gradient from the stomach to the cecum coincident with the migration of ENCCs towards the cecum [75,76]. The side of the cell body from which axons emerge and the initial direction of axon growth of some CNS neurons can be influenced by gradients of guidance factors [77,78]. We provide two lines of evidence to suggest that individual ENCCs cannot sense the polarity of the gut. First, our data suggest that the polarization of an ENCC cell body required for the initial formation of an axon is random, and that neurites must project for a minimum distance, or exist for a minimum amount of time, before they are able to detect the longitudinal axis of the gut. Second, the migration of solitary ENCCs was consistent with an unbiased random walk. This implies that individual ENCCs are unable to detect chemotactic gradients along the gut. In *Xenopus*, groups of cranial neural crest cells show a strong chemotactic response towards the chemokine, Sdf1, but single cells respond poorly and thus cell-cell interactions are essential for chemotaxis of cranial neural crest cells towards Sdf1 [9]. It would therefore be very interesting to determine whether solitary ENCCs show a chemotactic response to GDNF.

## Conclusions

ENCCs exhibit variable speeds and directionalities, but with a caudal bias, and this results in the population of each gut region as well as the caudal advance of some ENCCs. Hence, the gut is not populated by cells stopping, but by cells migrating non-directionally. ENCCs remain migratory after colonizing each gut region, which might be a mechanism to ensure an even density of cells as the gut grows. Despite the variability in behavior, ENCCs overall behave differently in groups compared to solitary cells and therefore meet the definition of collective migration [45]. Moreover, previous studies have shown that ENCCs can act cooperatively in the regulation of proliferation and migration [60,79]. Chains of ENCCs appear to advance caudally because they prefer regions with low ENCC density, whereas neurites can detect the longitudinal axis of the gut and exhibit directional growth, although they do not lead the way down the gut. Future studies are needed of other cell populations that exhibit directional dispersion to reveal if the behavior of ENCCs can be generalized.

## Methods

### Mice

*Ednrb-hKikGR* mice, in which all ENCCs express the photoconvertible protein, KikGR, [26] were used for all experiments. C57BL/6 mice were time-, plug-mated to *Ednrb-hKikGR* mice. Segments of small intestines from E11.5 *Ret*^TGM/TGM^ mice [80] were also used for some experiments. Mice carrying E11.5 or E12.5 embryos were killed by cervical dislocation. All experiments were approved by the Anatomy & Neuroscience, Pathology, Pharmacology and Physiology Animal Ethics Committee of the University of Melbourne.

### Time-lapse imaging and analysis

The guts from E11.5 or E12.5 *Ednrb-hKikGR* mice weres dissected in DMEM/F12 containing 20 mM glutamine, 10% FBS and penicillin/streptomycin, and attached across a “V” cut into a piece of filter paper as described previously [81]. The filter paper was inverted and placed in a 35-mm coverslip-bottomed dish. A stainless steel ring was placed on the edge of the filter paper support to prevent movement. In some experiments, the culture medium also contained the EDNRB antagonist, BQ-788 (Sigma-Aldrich, Castle Hill, NSW, Australia; 20 μM), which was added three to four hours prior to the commencement of imaging. Co-cultures were also set up in which segments of small intestine from E11.5 *Ednrb-hKikGR* mice were placed at both the rostral and caudal ends of segments of small intestine from *Ret*^TGM/TGM^ mice, which lack ENCCs [36]. The co-cultures were grown in an incubator at 37°C with 5% CO_2_ for 24 to 48 hours prior to imaging. Preparations to be imaged were placed in an environmental chamber, held at 37°C, mounted on an inverted Leica SP5 laser confocal microscope (North Ryde, NSW, Australia). Photoconversion of individual or groups of KikGR + cells was performed using a 405 nm laser and the regions of interest (ROI) function on the confocal microscope. *Z*-series images were obtained through the ENCC network at 2.5-, 3-, 4- or 5-minute intervals using X10, X20 or X40 oil immersion objectives. Cell tracking (manual and automatic), and measurements of angles and distances, were performed on videos of projected *z*-series using Axiovision (Zeiss, North Ryde, NSW, Australia) or ImageJ (National Institutes of Health, USA) software. Filopodial extensions were quantified by counting the number of new filopodial extensions outside of the ENCC network in a 100 × 200 μm area at five-minute intervals for three hours. Immunohistochemistry was performed using Tuj1 antibodies (Covance, North Ryde, NSW, Australia) as reported previously [39].

### Random walk analysis

To determine whether solitary ENCCs moved in a random walk, data describing the (*x*(*t*), *y*(*t*)) coordinates of 21 solitary ENCC trajectories (ENCCs that were not in contact with any other ENCCs for a minimum of two hours) were analyzed. First, to detect whether there was any bias in the motion, the time evolution of the displacement in the x-direction and y-direction was estimated by calculating

(1) $$X\left(t\right)=\left(x\left(t\right)-x\left(0\right)\right),Y\left(t\right)=\left(y\left(t\right)-y\left(0\right)\right).$$

We then fitted the observed *X*(*t*), *Y*(*t*) data to the following model

(2) $$X\left(t\right)=\nu_{x}t,Y\left(t\right)=\nu_{y}t.$$

This fitting procedure allowed us to estimate the drift velocities *v*_*x*_and *v*_*y*_. An unbiased random walk has *v*_*x*_ = *v*_*y*_ = 0. Our data suggest that the mean drift in the x-direction is *v*_*x*_ = 0.020 ± 0.062 μm/minute, and the mean drift in the y-direction is *v*_*y*_ = 0.020 ± 0.078 μm/minute. Here we have quantified the uncertainty in our estimates using the sample standard deviation. Since we only have a relatively small number of trajectories, the sample standard deviation is large. In summary, given the large variability in our data, our estimates of *v*_*x*_ and *v*_*y*_ are sufficiently close to zero to conclude that the motion is, on average, unbiased.

To determine whether the motion followed a standard unbiased random walk, we fitted a power-law to the time evolution of the square of the displacement

(3) $$r^{2}\left(t\right)=\beta t^{\alpha },$$

Where *r*^2^(*t*) = *X*^2^(*t*) + *Y*^2^(*t*), and α and β are positive constants; for a standard unbiased random walk, α = 1. To determine whether our data are consistent with an unbiased random walk, we fitted the data for each solitary ENCC trajectory to Equation (3) to give 21 estimates of α from which we could estimate an average value of this exponent. To fit our data, we rearranged Equation (3) to obtain

(4) $${\text{log}}_{10}\left(\frac{r^{2}\left(t\right)}{t}\right)={\text{log}}_{10}\left(\beta \right)+\left(\alpha -1\right){\text{log}}_{10}\left(t\right).$$

By fitting a straight line to a plot of ${\text{log}}_{10}\left(\frac{r^{2}\left(t\right)}{t}\right)$ as a function of log_10_(*t*), the least-squares estimate of the slope of that line provided us with an estimate of (*α* - 1).

## Abbreviations

EDNRB: endothelin receptor type B; ENCCs: enteric neural crest-derived cells; KBP: kinesin binding protein; PKA: protein kinase A.

## Competing interests

The authors declare that they have no competing interests.

## Authors’ contributions

HMY conceived the project, contributed to data collection and analyses, and drafted the manuscript. AJB and MMH contributed to data collection, analyses and experimental design. MJS performed random walk analysis. SJM contributed to the experimental design. CRA contributed to analyses and statistics. HE conceived the project. All authors read and approved the final manuscript.

## Supplementary Material

Additional file 1: Movie 1 Explant of colon from an E12.5 *Ednrb-hKikGR* mouse in which a group of ENCCs that were 600 to 700 μm from the wavefront were photoconverted from green to red. Some of the photoconverted ENCCs migrated along longitudinally-oriented strands with high speed (cells tracks indicated by blue and purple lines). *Z*-series images using a X10 objective lens through the ENCC network were captured every four minutes for 10 hours, and then each *z*-series projected. Caudal is to the right. Click here for file

Additional file 2: Movie 2 Same explant as shown in Movie 1. One of the photoconverted ENCCs exhibited a complex, circular pathway as indicated by the white line. This ENCC migrated at an average of 70 μm/h, but it advanced caudally only 140 μm after 16 hours. In the middle of the movie when it is migrating rostrally, it collides with a brighter green ENCC that is migrating caudally, but still proceeds rostrally. Caudal is to the right. Click here for file

Additional file 3: Movie 3 Explant of colon from an E12.5 *Ednrb-hKikGR* mouse. Red channel showing only four groups of photoconverted ENCCs, which were 80, 200, 600 and 1,000 μm from the most caudal cell at the commencement of imaging. ENCC do not retain their spatial order, and there is significant intermixing of cells photoconverted at different locations. Images were captured every 10 minutes for five hours using a X10 objective lens. Caudal is to the right. Click here for file

Additional file 4: Figure S1 Morphology of ENCCs and filopodia and lamellopodia extension. Figure S2: Interactions between the filopodia and/or lamellipodia at the front of one chain and cells or filopodia from another chain resulted in three general outcomes: adhesive, “walk-past” and repulsive (contact inhibition of locomotion). Figure S3: There were no significant differences in the number of Hu + neurons per area of Kik + cells in control explants (n = 12) and in explants cultured in the presence of BQ788 (20 μM) (n = 13) for 10 hours. At the end of the culture period, explants were fixed, processed for immunohistochemistry using an antibody to the pan-neuronal marker, Hu. The number of Hu + cells per area of Kik + cells was quantified using Fiji software. Click here for file

Additional file 5: Movie 4 Higher magnification movie showing a photoconverted ENCC in the middle of the field of view, which is the daughter cell of a cell that was photoconverted when it was undergoing mitosis; the nucleus is red and the cytoplasm is orange because of the presence of newly synthesized (green) protein after cell division. This ENCC, and other ENCCs in the field of view at the fronts of chains, extended filopodia and lamellopodia in a variety of directions. After the red ENCC encountered another chain, a number of processes adhered to the chain and the cell then joined the chain. The movement between images is due to spontaneous contractions of the developing external muscle. Images were captured every 2.5 minutes for five hours using a X40 objective lens for two hours. Caudal is to the right. Click here for file

Additional file 6: Movie 5 Red channel only after ENCCs 600 μm from the wavefront of the E12.5 colon had been photoconverted. Longitudinally projecting neurites are present on the caudal side. Images were captured every five minutes for 5 hours using a X20 objective lens for 9.5 hours. Caudal is to the right. Click here for file

Additional file 7: Movie 6 Movie in which all ENCCs, except those approximately 100 μm from the wavefront, were photoconverted from green to red. ENCCs use the red neurite in the middle of the field of view as a substrate to advance caudally. Images were captured using a X40 objective lens every four minutes for 16 hours, but this movie only shows a 3-hour period starting 11 hours after photoconversion and the commencement of imaging. The brightness of the green channel is reduced. Caudal is to the right. Click here for file
