# Supplementary material for: Colonizing while migrating: how do individual enteric neural crest cells behave?
Source: BMC Biol. 2014 Mar 26;12:23. doi: 10.1186/1741-7007-12-23 (PMC4101823; doi:10.1186/1741-7007-12-23)
Supplement: Additional file 4: Figure S1 — Morphology of ENCCs and filopodia and lamellopodia extension. Figure S2: Interactions between the filopodia and/or lamellipodia at the front of one chain and cells or filopodia from another chain resulted in three general outcomes: adhesive, “walk-past” and repulsive (contact inhibition of locomotion). Figure S3: There were no significant differences in the number of Hu + neurons per area of Kik + cells in control explants (n = 12) and in explants cultured in the presence of BQ788 (20 μM) (n = 13) for 10 hours. At the end of the culture period, explants were fixed, processed for immunohistochemistry using an antibody to the pan-neuronal marker, Hu. The number of Hu + cells per area of Kik + cells was quantified using Fiji software. [file 1741-7007-12-23-S4.docx]

**
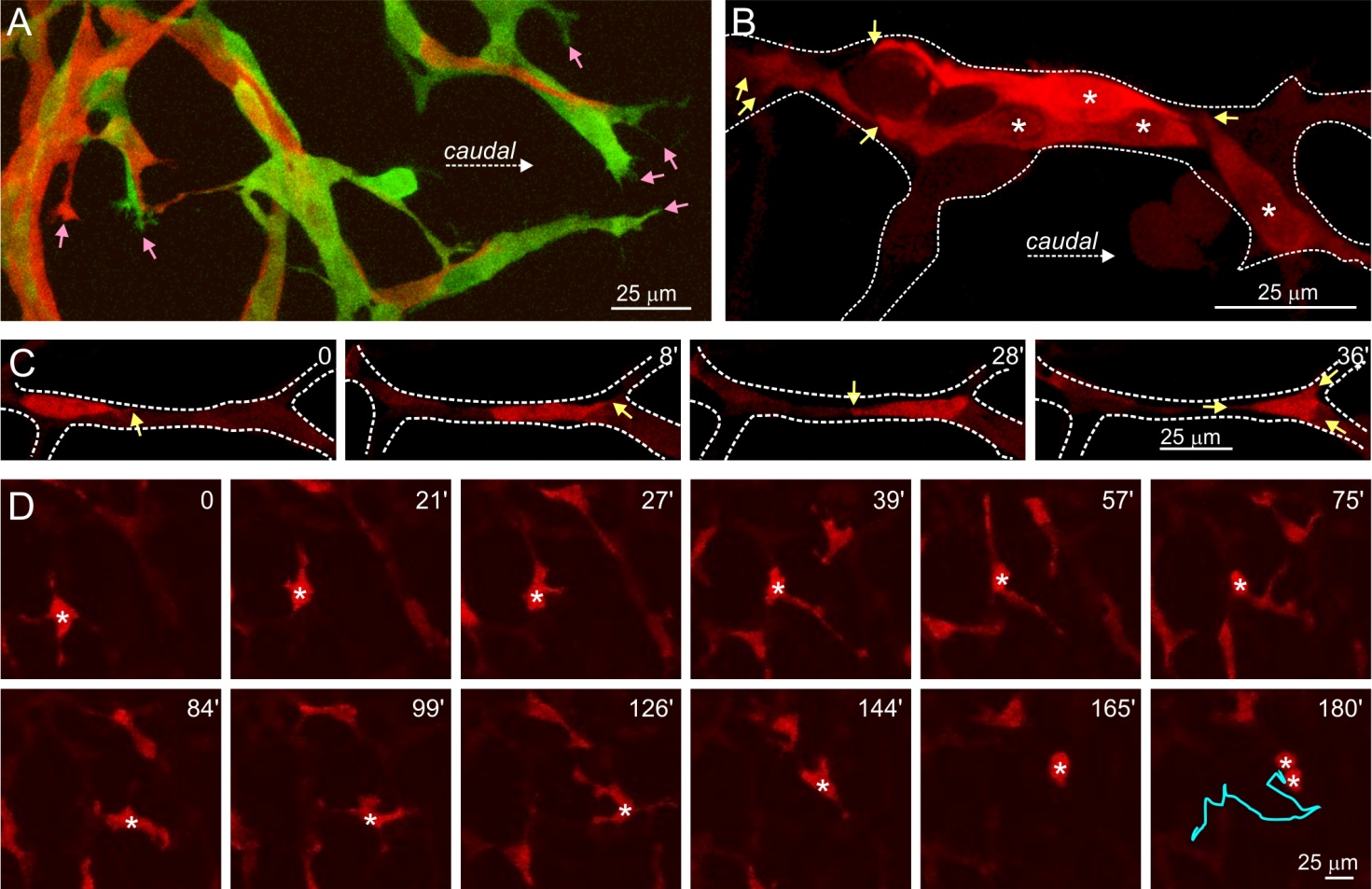
**

**Additional figure 1.** Morphology of ENCCs and filopodia and lamellopodia extension. **A.** Migratory wavefront of an explant that contains a mixture of red (photoconverted) and un-photoconverted (green) ENCCs. Cells at the fronts of chains extend filopodia and lamellopodia (*pink arrows*) in a variety of directions into ENCC-free spaces and then migrated into ENCC-free regions. **B.** Red channel only showing 4 photoconverted ENCCs (*asterisks*) in a chain close to the wavefront; the border of the chain is indicated by the dotted lines. The cells extend lamellopodia and filopodia within the network (*yellow arrows*). Cells at the front of cranial neural crest chains in the chick extend more lamellipodia and filopodia than trailing cells [2]. We were unable to compare quantitatively protrusive activity at the front of chains with that of cells within chains because we could not always identify all filopodia and lamellopodia within chains with certainty (due to less contrast in fluorescence between photoconverted cells and other cells within chains). **C.** Selected frames of a photoconverted ENCC, close to the wavefront. At times it is unipolar and extends processes (*yellow arrows*) only at the front (0, 8’), but later it also extends a process behind (28’); when it reaches a junction, processes explore both branches (36’). There was no significant difference in the number of new protrusions outside of the network between the different regions (200-400 μm from wavefront: 7.2±1.8 new protrusions/h/20,000μm^2^; 500-800 μm from wavefront: 12.2±1.1 protrusions/h/20,000μm^2^, *t*-test, *p*>0.05, n=4 explants for each).

**D.** Selected frames of a photoconverted ENCC (*asterisk*), which was only 150 μm behind the most caudal cell at the commencement of imaging. The cell extended processes in many different directions and then divided. The trajectory of the cell is shown in the last frame.

**
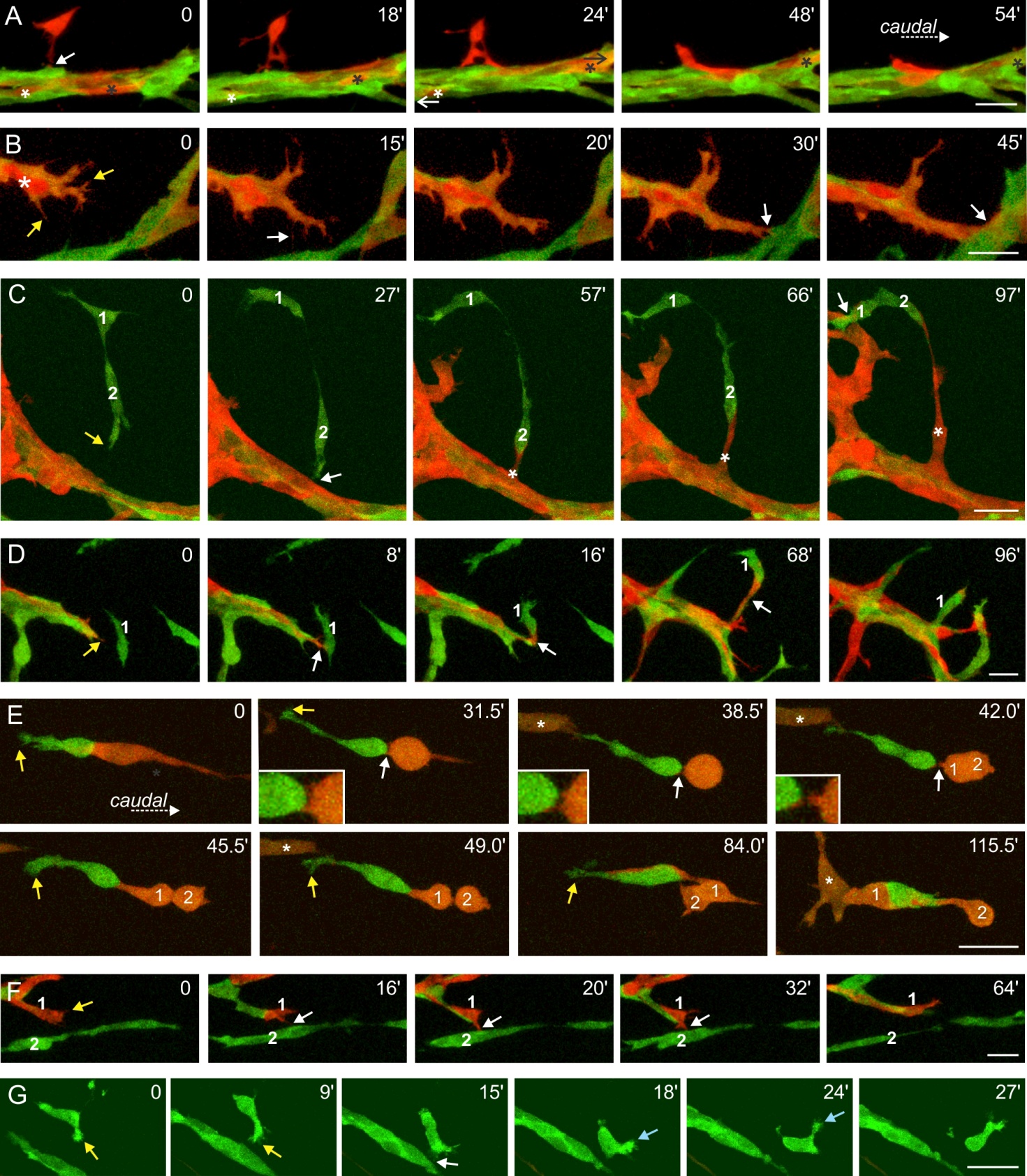
**

**Additional figure 2.** Interactions between the filopodia and/or lamellipodia at the front of one chain and cells or filopodia from another chain resulted in three general outcomes: adhesive, “walk-past” and repulsive (contact inhibition of locomotion). Most adhesive interactions between ENCCs resulted in ENCCs coalescing to form a single strand (**A-D**). Generally the smaller chain or a solitary cell joined the larger chain (**A**), but sometimes ENCCs exited thick strands following the interaction (**C**). Even when ENCCs rounded up to divide, they usually remained adhered to a neighbour (**E**). Second, some interactions were not adhesive or repulsive (**F**), which has been termed “walk-past” behavior in *Xenopus* [1]. Finally, behavior resembling contact inhibition of locomotion was also occasionally observed in which, following contact with another cell, ENCCs extended lamellopodia on the side away from the point of contact and changed migration direction (**G**). Interactions between two chains of ENCCs, or between a solitary ENCC and chain, all within 200 μm from the most caudal cell, were quantified; 86% of interactions were adhesive (95% confidence intervals = ±9.6%), 8% were walk-past* (95% confidence intervals = ±7.5%), and 6% of interactions were classified as contact inhibition of locomotion** (95% confidence intervals = ±6.6%) in that, following contact, one or both ENCC changed direction (50 interactions were analyzed in 14 explants). **A.** A process of a solitary photoconverted ENCC (red) encountered a thick chain of ENCCs. The solitary ENCC extended additional processes that adhered to the chain, and the cell then joined the chain. One of the cells within the chain (*white asterisk*) migrated rostrally out of the field of view, while another cell migrated caudally (*black asterisk*). **B.** High magnification images of the daughter cell of a cell that was photoconverted when it was undergoing mitosis; the nucleus (*asterisk*) is red whereas the cytoplasm is orange because of the presence of newly synthesized (green) protein after cell division. This cell was at the front of a chain and it extended filopodia and lamellopodia in a variety of directions (*yellow arrows*). After encountering another chain (15’), a number of processes adhered to the chain (*white arrows*) and the cell then joined the chain. **C.** Two green cells on their own extend processes (*yellow arrow*). When one of the processes of cell #2 contacts a chain, it adheres to one of the red cells in the chain (*white arrow*, 27’). The red cell (*white asterisk*) then leaves the chain adhered to the green cell (57’-97’). In the meantime, green cell #1 adhered to another chain (*white arrow*, 97’). **D.** A red cell at the front of a chain extends filopodia (*yellow arrow*), which contact a solitary green cell (1) and adhere to it (*white arrows*, 8’, 16’, 68’). The green arrow continues in the same direction for around 50 min, which draws the red cell out of the chain; later the green cell joins the chain (96’). **E.** Two cells, one red and one green, which had earlier broken off a chain. Both cells extend processes away from the point of contact (*yellow arrows*). The red cell rounded up to divide (31.5’), and even when completely round (38.5’), and immediately after division (42.0’), adhesions with the green cell were visible (*white arrows*). At 115.5’, the group of 3 cells encountered the lead cell in another chain (*asterisk*) and adhered. **F.** An example of walk-past behavior: Processes extend caudally from a red cell (#1) at the front of a chain (*yellow arrows*). The processes contact (*white arrow*s) a green cell (#2), which is part of a slender chain of cells, but no adhesion occurs; cell #1 continues migrating in a similar direction to that in which it was migrating prior to interacting with the second chain of cells (64’). **G.** Processes extend from a green cell (yellow arrows), and then contact another cell (*white arrow*). Following the contact, the cell extends processes from the opposite side (*blue arrows*) and migrates away from the point of contact.

*”Walk-past” was defined as interactions in which the change in angle of migration following contact with another ENCC was <45°; in the 4 walk-past interactions analyzed, the changes in migration angle 30 minutes after contact with another ENCC compared to that 30 minutes before contact were 11°, 11°, 4° and 22°. Angles were measured at 4 minute intervals and then the average pre-contact angle compared to the average post-contact migration angle.

** Contact inhibition of locomotion was defined as interactions in which the change in angle of migration following contact with another ENCC was >45°; in the 3 interactions classified as contact inhibition of locomotion, the differences in migration angle before and after contact with another ENCC were 73°, 203°, and 111°.


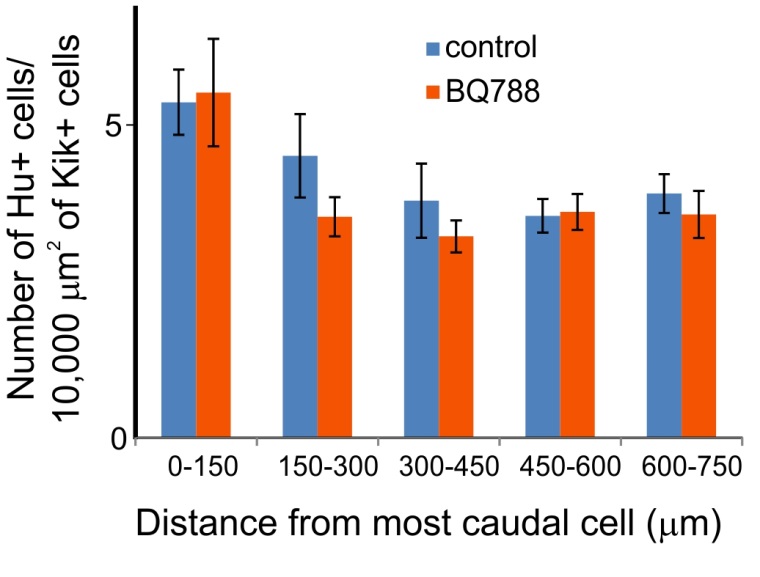


**Additional figure 3.** There were no significant differences in the number of Hu+ neurons per area of Kik+ cells in control explants (n = 12) and in explants cultured in the presence of BQ788 (20 μM) (n = 13) for 10 hours. At the end of the culture period, explants were fixed, processed for immunohistochemistry using an antibody to the pan-neuronal marker, Hu. The number of Hu+ cells per area of Kik+ cells was quantified using Fiji software.

**References**

1. Scarpa E, Roycroft A, Theveneau E, Terriac E, Piel M, Mayor R: **A novel method to study contact inhibition of locomotion using micropatterned substrates.** *Biol Open* 2013, **2**:901-906.

2. Teddy JM, Kulesa PM: **In vivo evidence for short- and long-range cell communication in cranial neural crest cells.** *Development* 2004, **131**:6141-6151.

**Movie Descriptions**

**Movie 1:** Explant of colon from an E12.5 *Ednrb-hKikGR* mouse in which a group of ENCCs that were 600-700 μm from the wavefront were photoconverted from green to red. Some of the photoconverted ENCCs migrated along longitudinally-oriented strands with high speed (cells tracks indicated by blue and purple lines). *Z*-series images using a X10 objective lens through the ENCC network were captured every 4 minutes for 10 hours, and then each *z*-series projected. Caudal is to the right.

**Movie 2**: Same explant as shown in Movie 1. One of the photoconverted ENCCs exhibited a complex, circular pathway as indicated by the white line. This ENCC migrated at an average of 70 μm/h, but it advanced caudally only 140 μm after 16 hours. In the middle of the movie when it is migrating rostrally, it collides with a brighter green ENCC that is migrating caudally, but still proceeds rostrally. Caudal is to the right.

**Movie 3:** Explant of colon from an E12.5 *Ednrb-hKikGR* mouse. Red channel only showing 4 groups of photoconverted ENCCs, which were 80, 200, 600 and 1000 μm from the most caudal cell at the commencement of imaging. ENCC do not retain their spatial order, and there is significant intermixing of cells photoconverted at different locations. Images were captured every 10 minutes for 5 hours using a X10 objective lens. Caudal is to the right.

**Movie 4:** Higher magnification movie showing a photoconverted ENCC in the middle of the field of view, which is the daughter cell of a cell that was photoconverted when it was undergoing mitosis; the nucleus is red and the cytoplasm is orange because of the presence of newly synthesized (green) protein after cell division. This ENCC, and other ENCCs in the field of view at the fronts of chains, extended filopodia and lamellopodia in a variety of directions. After the red ENCC encountered another chain, a number of processes adhered to the chain and the cell then joined the chain. The movement between images is due to spontaneous contractions of the developing external muscle. Images were captured every 2.5 minutes for 5 hours using a X40 objective lens for 2 hours. Caudal is to the right.

**Movie 5:** Red channel only after ENCCs 600 μm from the wavefront of the E12.5 colon had been photoconverted. Longitudinally projecting neurites are present on the caudal side. Images were captured every 5 minutes for 5 hours using a X20 objective lens for 9.5 hours. Caudal is to the right.

**Movie 6:** Movie in which all ENCCs, except those ~100 μm from the wavefront, were photoconverted from green to red. ENCCs use the red neurite in the middle of the field of view as a substrate to advance caudally. Images were captured using a X40 objective lens every 4 minutes for 16 hours, but this movie only shows a 3 hour period starting 11 hours after photoconversion and the commencement of imaging. The brightness of the green channel is reduced. Caudal is to the right.
